# Subclinical Hearing Loss, Longer Sleep Duration, and Cardiometabolic Risk Factors in Japanese General Population

**DOI:** 10.1155/2014/218218

**Published:** 2014-08-12

**Authors:** Kei Nakajima, Eiichiro Kanda, Ami Hosobuchi, Kaname Suwa

**Affiliations:** ^1^Division of Clinical Nutrition, Department of Medical Dietetics, Faculty of Pharmaceutical Sciences, Josai University, 1-1 Keyakidai, Sakado, Saitama 350-0295, Japan; ^2^Department of Nephrology, Tokyo Kyosai Hospital, Nakameguro 2-3-8, Meguroku, Tokyo 153-8934, Japan; ^3^Saitama Health Promotion Corporation, 519 Kamiookubo, Saitama, Saitama 338-0824, Japan

## Abstract

Hearing loss leads to impaired social functioning and quality of life. Hearing loss is also associated with sleeping disorders and cardiometabolic risk factors. Here, we determined whether subclinical hearing loss is associated with sleep duration and cardiometabolic risk factors in a cross-sectional and longitudinal study of healthy Japanese general population. 48,091 men and women aged 20–79 years who underwent medical checkups were included in a cross-sectional study, and 6,674 were included in an 8-year longitudinal study. The prevalence of audiometrically determined hearing loss (>25 dB) at 4000 and 1000 Hz increased significantly with increasing sleep duration in any age strata. Logistic regression analysis showed that compared with reference sleep duration (6 h) longer sleep duration (≥8 h) was significantly associated with hearing loss, even after adjusting for potential confounding factors. Simultaneously, hearing loss was significantly associated with male sex, diabetes, and no habitual exercise. In the longitudinal study, the risk of longer sleep duration (≥8 h) after 8 years was significantly greater in subjects with hearing loss at 4000 Hz at baseline. In conclusion, current results suggest a potential association of subclinical hearing loss with longer sleep duration and cardiometabolic risk factors in a Japanese general population.

## 1. Introduction 

The progressive aging of society is leading to an increase in the prevalence of hearing loss worldwide. Although hearing loss is not directly life threatening, it may impair social functioning and quality of life, causing isolation, frustration, and impaired communication [1–6]. Meanwhile, several studies have revealed that sleeping disorders such as insomnia and daytime sleepiness are associated with hearing impairments, including hearing loss and tinnitus [7–10]. Therefore, some factors associated with sleep may be associated with hearing loss. To date, however, no study has examined the putative association between hearing loss and sleep duration.

In this context, we focused on subclinical objective hearing loss, which is often undetected and left untreated [4, 6], and investigated the lifestyles of individuals with subclinical hearing loss and the etiology of subclinical hearing loss. Because frequency of 500 to 4000 Hz is important range for speech processing [6], we determined whether hearing function at representative high (4000 Hz) and low (1000 Hz) frequencies, which are usually examined in a hearing screening test in Japan [11–13], was associated with lifestyle factors, including sleep duration per night and cardiometabolic risk factors, in a cross-sectional study of Japanese general population.

Because hearing loss has been shown to be associated with cardiometabolic risk factors, such as diabetes and smoking [11–16], we considered these factors as relevant confounding factors and also examined the associations between hearing loss and these cardiometabolic risk factors. To examine the effects of subclinical hearing loss on the incidence of longer sleep duration (8 h and ≥9 h), we performed a retrospective 8-year longitudinal study in an independent group of subjects whose sleep duration was classified as normal or short (≤7 h) at baseline.

## 2. Methods

### 2.1. Study Design

This study was based on a composite research program that is being conducted to identify the factors associated with cardiometabolic and atherosclerotic diseases. The design of this study is described in more detail elsewhere [17]. This retrospective study consists of data recorded during annual medical checkups of asymptomatic individuals living or working in Saitama Prefecture, a suburb of Tokyo, Japan. The study started in 2011 and involves the collaboration of three institutions in Saitama: Jichi Medical University, Josai University, and Saitama Health Promotion Corporation. The protocol, which conforms to the Declaration of Helsinki, was approved by the Ethics Committee of Jichi Medical University and Josai University and by the Committee of the Saitama Health Promotion Corporation. Written informed consent was obtained from all participants. Since 1997, Saitama Health Promotion Corporation, a public interest corporation, has supported the health of individuals, including children and adolescents, living or working in Saitama Prefecture, primarily by carrying out various types of medical checkups [18].

### 2.2. Subjects

#### 2.2.1. Cross-Sectional Study

**Figure 1:**
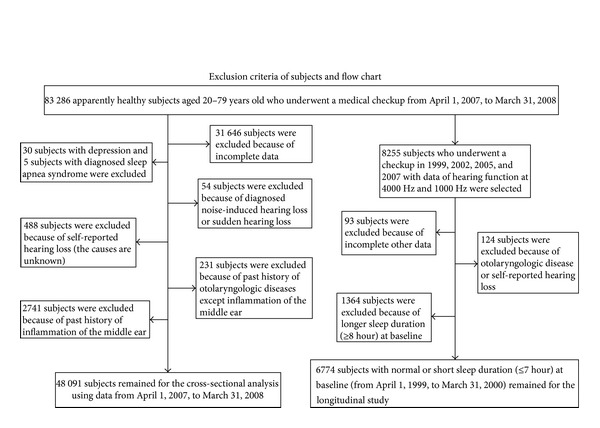
Exclusion criteria and subject disposition.

We digitally stored data from 83 286 apparently healthy subjects aged 20–79 years old who underwent medical checkups at Saitama Health Promotion Corporation between April 1, 2007, and March 31, 2008. Subjects with diagnosed or undiagnosed self-reported hearing loss (*n* = 488) were excluded from the analysis because the cause and treatment received (e.g., hearing aids and pharmacotherapy) were not available in this study. Subjects with self-reported depression and sleep apnea syndrome were also excluded because these conditions may affect sleep duration [19–22]. The exclusion criteria applied in this study and the disposition of subjects are shown in Figure 1. Subjects with self-reported tinnitus (*n* = 1, 396) were included because tinnitus was usually mild and was not always diagnosed by a physician in this study. Consequently, 48 091 subjects were included in the cross-sectional study.

#### 2.2.2. Longitudinal Study

When we selected subjects for the longitudinal study from those included in the cross-sectional study, the number of subjects whose baseline sleep duration was normal or short (≤7 h) and who underwent the same checkup four times between April 1, 1999, and March 31, 2008 (8 years duration), was <1,000. Therefore, subjects included in the longitudinal study were identified from the original study population, which means the subjects included in the longitudinal study differed from those included in the cross-sectional study. However, the assessment of hearing loss and other laboratory tests were identical between the cross-sectional and longitudinal studies. After excluding subjects with incomplete data and those with known ear diseases, 6,774 subjects with normal or short sleep duration at baseline were included in the longitudinal study (Figure 1). During 8 years, many subjects abandoned the medical checkup held by Saitama Health Promotion Corporation (average proportions of not undergoing the same checkup next year was approximately one-fourth to one-third during 1999~2008) and changed to other checkups because of house moving, resignation, or change of jobs, child care, or family reasons, resulting in the decreased number of subjects in the longitudinal study.

#### 2.2.3. Anthropometric, Laboratory, and Audiometric Tests

Anthropometric, laboratory, and audiometric tests were carried out in the morning. Serum parameters were measured using standard methods on Hitachi autoanalyzers (Tokyo, Japan) at Saitama Health Promotion Corporation. Hemoglobin (Hb) A1c was converted to national glycohemoglobin standardization program levels using a validated formula [23]. Unfortunately, fasting plasma glucose levels were not available in both studies. The hearing test was conducted in a quiet room by trained staff using an ordinary audiometer. Subclinical (objective) hearing loss was defined as a pure-tone average hearing loss of >25 dB at high (4000 Hz) and low (1000 Hz) frequencies.

#### 2.2.4. Sleep Duration and Confounding Factors

Self-reported sleep duration per night, which was obtained as a response to the simple question about sleep, was divided into five categories (≤5, 6, 7, 8, and ≥9 h) according to previous studies [24, 25]. The duration of daytime nap was not taken into consideration in this study. Subjects completed a form to record history of cardiovascular disease (including stroke), complications (hypertension, diabetes, or dyslipidemia), alcohol consumption (no, occasional, 1–3 times/week, 4–6 times/week, or daily), smoking status (no, past, or current), regular exercise (≥30 min per time; no, occasional, once/week, or at least twice/week), and work duration (≤6, 7, 8, 9, 10, or ≥11 h). The influence of body weight was evaluated in terms of body mass index (BMI), which was divided into six categories (≤18.9, 19.0–20.9, 21.0–22.9, 23.0–24.9, 25.0–26.9, and ≥27.0 kg/m^2^). We took into consideration that WHO has proposed that BMI cutoff points for overweight and obesity for Asian populations should be lower (≥23.0 kg/m^2^ and ≥27.5 kg/m^2^, resp.) compared to Western populations [26]. Since the proportions of subjects classified as underweight (i.e., <18.5 kg/m^2^) or obese (i.e., ≥30.0 kg/m^2^) are very low (4.8% and 5.0%, resp.) in this study, we round up the low and high BMI cutoffs to 19 and 27 kg/m^2^ (7.4% and 15.1%, resp.). The influence of systemic inflammation was roughly evaluated in terms of the circulating white blood cell count, a putative risk factor for cardiovascular disease [27–29], which was divided into quartiles. Because organic solvents can affect sleep duration [30, 31], the use of organic solvent in the workplace was taken into account, although the type was not recorded and the number of subjects was limited (*n* = 39, 691).

#### 2.2.5. Statistical Analysis

Data are expressed as means (SD) or medians (interquartile range). Differences in clinical parameters and categorical variables between the five categories of sleep duration were examined by one-way analysis of variance (ANOVA) and *χ* ^2^ tests, respectively. The prevalence of hearing loss was first evaluated in four age groups (20–39, 40–49, 50–51, and 60–79 years) because advancing age is one of the main risk factors for hearing loss. In the cross-sectional study, multivariate logistic regression models were used to examine whether subclinical hearing loss was associated with lifestyle and cardiometabolic risk factors to calculate odds ratios (OR) and 95% confidence intervals (CI) with adjustment for relevant confounders. After sleep durations in five categories of sleep duration were coded as 5, 6, 7, 8, and 9 for ≤5 h, 6 h, 7 h, 8 h, and ≥9 h, respectively, associations between sleep duration as a continuous variable and hearing loss were examined. The associations between relevant confounders and hearing loss were also evaluated.

For the longitudinal analysis, multivariate logistic regression models were also used to examine the association between baseline hearing loss and risk of longer sleep duration (8 h and ≥9 h) after 9 years to calculate relative risk (RR) and 95% confidence interval (CI) because the incidence of longer sleep duration after 9 years was <10% and the ORs are expressed as relative risks in the longitudinal study [32]. In this analysis, the provisional reference BMI categories were defined as a BMI of 21.0–22.9 kg/m^2^ based on the proposal by the World Health Organization regarding overweight and obese classifications for Asian populations [26]. Statistical analyses were performed using IBM-SPSS version 18.0 (PASW statistics 18; Chicago, IL, USA) and Statview version 5.0 (SAS Institute; Cary, NC, USA). Values of *P* < 0.05 were considered statistically significant.

## 3. Results

### 3.1. Cross-Sectional Study

The clinical characteristics of the subjects are presented in Table 1. Subjects with longer sleep duration were more frequently men, were older, and had a shorter work duration, with increased numbers of cardiometabolic risk factors (especially decreased high-density lipoprotein cholesterol). BMI, white blood cell count, and the frequency of current smokers, no regular exercise, or use of organic solvents at work showed U- or J-shaped relationships with sleep duration. The prevalence of cardiovascular disease, hypertension, and diabetes increased with increasing sleep duration. Similarly, the prevalence of hearing loss increased with increasing sleep duration, irrespective of the left or right ear, or frequency. The proportions of manufacturing and construction workers were higher in the longer sleep duration groups (8 h and ≥9 h), whereas the opposite was true for the proportions of clerical, technical, and medical workers.

**Figure 2:**
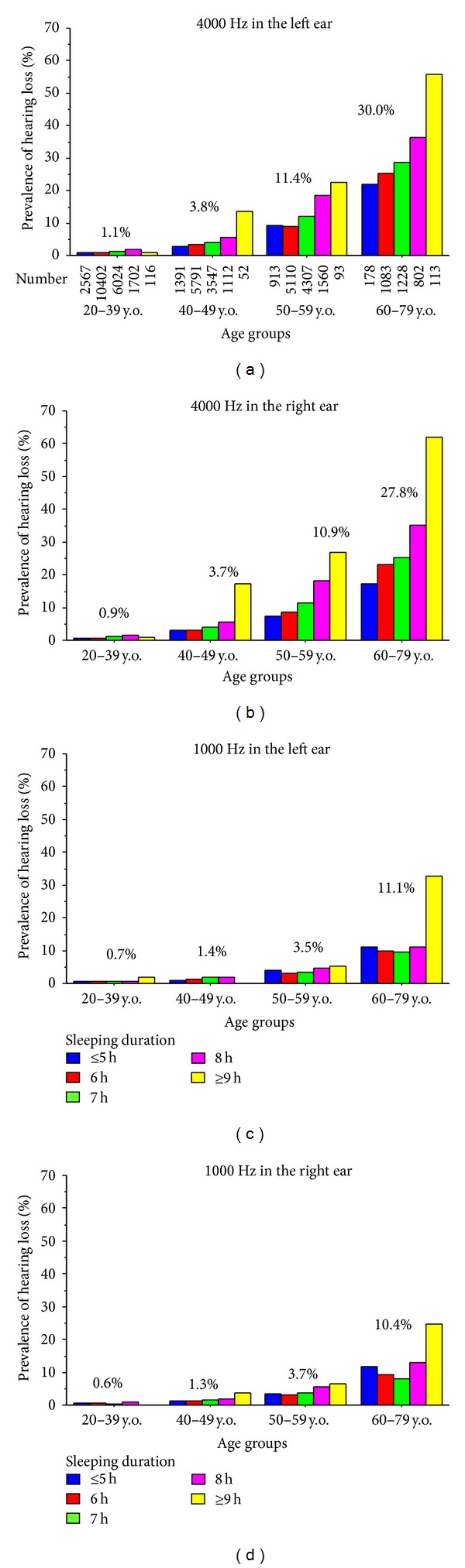
Prevalence of hearing loss according to age group. The number of subjects is shown under the column for hearing loss at 4000 Hz in the left ear (top-left panel). The numbers of subjects included in the other panels were identical. The percentage (%) above the column expresses the average of prevalence of hearing loss according to the age group. The prevalence of hearing loss at 4000 Hz, but not at 1000 Hz, increased significantly with increasing sleep duration in most age groups (*χ* ^2^ test). Left ear at 4000 Hz: 20–39 years, *P* = 0.002; all other age groups, *P* < 0.001. Right ear at 4000 Hz: all age groups, *P* < 0.001. Left ear at 1000 Hz: 20–39 years, *P* = 0.51; 40–49 years, *P* = 0.20; 50–59 years, *P* = 0.08; 60–79 years, *P* < 0.001. Right ear at 1000 Hz: 20–39 years, *P* = 0.34; 40–49 years, *P* = 0.13; 50–59 years, *P* < 0.001; 60–79 years, *P* < 0.001. y.o.: years old.

Figure 2 shows the prevalence of hearing loss according to age groups. The prevalence of hearing loss at 4000 Hz increased significantly with increasing sleep duration in any age strata except for younger age (20–39 years old). Likewise, the prevalence of hearing loss at 1000 Hz increased significantly with increasing sleep duration, particularly in the older age groups. However, there seemed to be a slight J-shaped relationship in subjects aged 60–79 years.

Multivariate logistic regression analyses showed that, compared with sleep duration of ≤5 h, the other categories of sleep duration were significantly associated with hearing loss at 4000 and 1000 Hz at least in one ear (Table 2). Adjustment for confounding factors, including age and sex, markedly attenuated the associations, but changing the reference sleep duration category from ≤5 h to 6 h (Model 2b) or additional adjustment for the use of organic solvents at work (Model 3) did not. Likewise, sleep duration as a continuous variable was also significantly associated with hearing loss at 4000 and 1000 Hz.

Table 3 shows the ORs for the confounding factors, except age, for hearing loss. Male sex, diabetes, no regular exercise, and tinnitus were significantly associated with hearing loss at both frequencies, whereas regular alcohol consumption, but not daily alcohol consumption, was inversely associated with hearing loss. Low body weight, the highest quartile of white blood cell count (≥88.9 ×10^2^/*μ*L), current smoking, and daily alcohol consumption were significantly associated with hearing loss at 4000 Hz. Work duration was significantly and inversely associated with hearing loss at 1000 Hz but not at 4000 Hz. Compared with clerical work, other work types, except managerial and medical work, were significantly associated with hearing loss at 4000 Hz (ORs 1.24–4.49) and 1000 Hz (ORs 1.34–1.90), even after controlling for confounding factors, including work duration (data not shown).

### 3.2. Longitudinal Study

The baseline clinical characteristics of subjects included in the longitudinal study (Table 4) are similar to those of the subjects included in the cross-sectional study (Table 1).  Table 5 shows RRs of hearing loss at baseline for the longer sleep duration (≥8 hr) after 9 years. The risk of long sleep duration was significantly greater in subjects with hearing loss at 4000 Hz at baseline compared with subjects without such hearing loss. This increased risk remained significant after adjusting for potential confounding factors at baseline. Although subjects with hearing loss at 1000 Hz at baseline had a significantly higher risk for longer sleep duration, this disappeared after adjusting for fundamental confounding factors (Model 2).

## 4. Discussion

Our cross-sectional and longitudinal studies provide robust evidence that subclinical hearing loss, especially at high frequencies, is associated with longer sleep duration, which was particularly depicted in older age, independently of relevant lifestyle factors, cardiometabolic risk factors, and the use of organic solvents at work. These associations were not weakened when the reference sleep duration was changed to 6 h, the major sleep duration category in this study. Although several epidemiological studies have shown U- or J-shaped relationships between sleep duration and clinical disorders, such as obesity and diabetes [24, 33, 34], the relationship between hearing loss at 4000 Hz and sleep duration in our study was nearly linear in all of the age categories. Because many studies have shown that noise-induced hearing loss, a major form of acquired hearing loss [35], gradually begins around the frequency of 4000 Hz [36, 37], currently observed hearing loss might be attributable to a great extent to the long-term noise exposure, which was not measured in this study.

Regarding the cause-effect relationship, considering the results of our longitudinal study, it is likely that longer sleep duration may occur because of or in conjunction with hearing loss. In other words, subclinical hearing loss may be a predictor of long sleep duration, which may be related to the development of cardiometabolic disease [24, 33–38]. Hearing loss is also associated with dementia and cognitive impairment [39–42], whereas cognitive impairments, including Alzheimer disease, are associated with sleep and circadian problems [43–45]. Therefore, it is possible that long sleep duration might reflect other clinical conditions that were not examined in this study and that the currently observed associations may be spurious and unknown factors might mediate two conditions. Frustration and uneasiness associated with hearing loss or noisy daytime environments may reduce sleep quality and provoke insomnia [9, 10, 46] and may result in longer sleep durations. By contrast, once individuals with hearing loss do fall asleep, they could maintain good sleep without waking during the night. Hearing loss might also protect sleep by reducing the subjects' awareness of environmental noise during sleep [8] and might prolong sleep duration [46–48].

Meanwhile, many previous studies have provided evidence that diabetes, cardiometabolic disease, and smoking are associated with hearing loss [11–16], plausibly through diabetic micro- and macroangiopathy [14, 49, 50]. Thus, like typical diabetic complications, hearing loss may be one of complications following diabetic or atherosclerotic etiologies. Nevertheless, there is also a possibility that long-time sleep in turn may deteriorate the pathophysiology of diabetes and atherosclerosis because of putative associations between long-time sleep and diabetes [24, 33–38]. Intriguingly, low BMI (i.e., low body weight rather than obesity) was associated with hearing loss at 4000 Hz. Underweight might be associated with increased risk for hearing loss through inadequate intake of dietary nutrients, especially vitamin B_12_ and antioxidants [51–55], or other factors that were previously reported to be associated with increased mortality in underweight people [56–58]. In our study, hearing loss at 4000 Hz was also associated with current smoking, daily alcohol consumption, and high white blood cell count, which are all generally classified as cardiovascular risk factors [27–29, 59–62]. Recently, Ronksley et al. [60] have shown that neutrophil-lymphocyte ratio, white blood cell count, and C-reactive protein may be associated with hearing loss of diabetic patients. Taken together, although noise-induced hearing loss commonly begins at frequency of 4000 Hz [35–37], our results demonstrates that some cardiovascular risk factors might aggravate the pathophysiology of such noise-induced hearing loss. Then, long-term noise exposure and cardiovascular risk factors might provoke and aggravate a pivotal health damage of hearing loss and sequentially sleep disorder, which needs to be confirmed in further study.

## 5. Limitations

Several limitations should be mentioned. First, we assessed self-reported sleep duration but not the quality of sleep or other related factors. Nocturnal awakening, insomnia, nocturia, daytime napping, and difficulties with falling sleep may interfere with the observed associations [8, 63, 64]. Future studies should also include objective assessments of sleep duration, preferably made using actigraphy, because such factors may be important confounding factors. Second, we did not assess socioeconomic status in terms of education or annual income. It is possible that the site of residence, work duration, or shift work could shift the endogenous sleeping duration to an exogenously restricted sleeping duration [41, 65–67]. However, adjustment for the type or duration of work did not markedly alter the associations. Third, subclinical hearing loss was only determined using an audiometric test. Other screening methods, such as whispered voice, finger rubbing, and watch tick tests, can detect subclinical hearing loss that may not be identified with audiometric tests [6, 68]. Finally, our results may not be applicable to other populations who have different sleep durations, socioeconomic status, morbidities, and longevities, because these are likely to affect both sleep duration and subclinical hearing loss.

## 6. Conclusion

Our results suggest that subclinical hearing loss, especially at a high frequency, was independently associated with longer sleep duration and cardiometabolic risk factors in Japanese general population. Current findings remain to be warranted in further study.

## Figures and Tables

**Table 1:** Clinical characteristics of subjects according to the five sleep duration categories.

Five categories by sleep duration	Total	≤5 h	6 h	7 h	8 h	≥9 h
*N*, (% of total)	48,091	5,049 (10.5)	22,386 (46.5)	15,106 (31.4)	5,176 (10.8)	374 (0.8)
Age, years	42.3 (12.1)	39.7 (11.2)	41.0 (11.7)	43.4 (12.3)	46.3 (12.8)	49.1 (15.5)
Men, *n* (%)	33,026 (68.7)	3,208 (63.5)	14,793 (66.1)	10,767 (71.3)	3,965 (76.6)	293 (78.3)
BMI, kg/m^2^	23.5 (3.6)	23.7 (3.9)	23.5 (3.7)	23.4 (3.4)	23.5 (3.5)	23.6 (3.5)
Systolic blood pressure, mmHg	123 (17.3)	121 (16.7)	122 (16.9)	124 (17.5)	127 (18.4)	128 (18.5)
Diastolic blood pressure, mmHg	75 (13.3)	74 (13.5)	74 (13.1)	75 (13.5)	77 (13.3)	77 (13.7)
Total cholesterol, mg/dL	199 (35.5)	198 (35.7)	199 (35.5)	199 (35.2)	201 (35.7)	198 (38.8)
Triglyceride, mg/dL	96 (64–153)	88 (59–146)	93 (62–148)	100 (67–157)	107 (70–170)	103 (73–169)
HDL cholesterol, mg/dL	61.4 (15.5)	61.9 (15.6)	61.5 (15.6)	61.2 (15.4)	61.1 (15.9)	59.0 (15.1)
HbA1c, %, NGSP	5.5 (0.7)	5.5 (0.8)	5.5 (0.7)	5.5 (0.7)	5.6 (0.8)	5.7 (1.1)
White blood cell, ×10^2^/*μ*L	65.2 (17.6)	66.5 (18.5)	64.8 (17.4)	65.0 (17.6)	66.3 (18.1)	67.3 (19.4)
Past history of CVD, *n* (%)	711 (1.5)	72 (1.4)	301 (1.3)	229 (1.5)	100 (1.9)	9 (2.4)
Complication						
Hypertension, *n* (%)	3,697 (7.7)	264 (5.2)	1,436 (6.4)	1,325 (8.8)	623 (12.0)	49 (13.1)
Dyslipidemia, *n* (%)	2,187 (4.5)	164 (3.2)	969 (4.3)	756 (5.0)	280 (5.4)	18 (4.8)
Diabetes, *n* (%)	1,402 (2.9)	106 (2.1)	580 (2.6)	477 (3.2)	217 (4.2)	22 (5.9)
Subjects with high HbA1c (≥6.5%), *n* (%)	2,521 (5.2)	217 (4.3)	1,019 (4.6)	851 (5.6)	397 (7.7)	37 (9.9)
Alcohol consumption						
1~3/w/4~6/w/daily (%)	16.7/11.6/16.9	17.9/8.1/12.6	17.7/10.9/13.7	15.9/13.1/19.7	14.3/13.9/26.4	11.0/8.8/30.2
Smoker						
Past/current (%)	11.2/38.1	6.8/40.5	10.4/36.9	12.9/37.6	13.8/41.7	10.7/44.7
Having regular exercise						
No/occasional~1/w/≥2/w (%)∗	51.0/31.8/17.2	57.4/28.4/14.3	51.4/32.2/16.4	48.4/33.2/18.4	49.9/30.2/20.0	57.0/24.9/18.2
Self-reported tinnitus, *n* (%)	1,396 (2.9)	121 (2.4)	635 (2.8)	457 (3.0)	170 (3.3)	13 (3.5)
Hearing loss at 4,000 Hz in the left ear, *n* (%)	3,068 (6.4)	188 (3.7)	1,013 (4.5)	1,100 (7.3)	675 (13.0)	92 (24.6)
Hearing loss at 4,000 Hz in the right ear, *n* (%)	2,883 (6.0)	158 (3.1)	952 (4.3)	1,012 (6.7)	656 (12.7)	105 (28.1)
Hearing loss at 1,000 Hz in the left ear, *n* (%)	1,097 (2.3)	90 (1.8)	404 (1.8)	367 (2.4)	192 (3.7)	44 (11.8)
Hearing loss at 1,000 Hz in the right ear, *n* (%)	1,070 (2.2)	81 (1.6)	390 (1.7)	338 (2.2)	225 (4.3)	36 (9.6)
Work duration, hour	8.6 (1.4)	9.2 (1.6)	8.7 (1.4)	8.4 (1.3)	8.3 (1.3)	8.1 (1.5)
Occupation, (%)∗						
Clerical workers	21.2	17.5	22.4	22.5	17.0	7.2
Production workers	8.3	6.7	7.4	9.4	9.9	12.8
Service workers	10.6	11.7	10.8	9.8	10.2	15.8
Managerial workers	6.8	5.9	6.5	7.7	6.9	2.9
Technical workers	5.2	6.6	5.4	4.8	4.2	2.7
Construction workers	6.6	5.7	5.4	6.6	12.2	20.3
Medical workers	4.4	4.9	4.6	4.2	3.9	2.9
Transport workers	3.1	6.8	2.7	2.2	3.4	6.2
Workers not classifiable or nonemployed	33.8	34.1	34.8	32.9	32.4	29.1
Organic solvent workers, *n* (%) (total available *n* = 39,691)	1,053 (2.7)	109 (2.6)	427 (2.3)	371 (3.0)	134 (3.2)	12 (4.0)

The data are expressed as means (SD). Triglyceride is expressed as medians (interquartiles).

∗Total sum is not 100% in some cases because of round-off to two decimal places.

Differences in parameters and categorical values between five sleep duration categories were significant (analysis of variance/*χ* ^2^ test; past history of CVD, and medical workers, both *P* = 0.01 and all others, *P* < 0.001), except self-reported tinnitus (*P* = 0.06).

BMI: body mass index, HDL: high-density lipoprotein, NGSP: national glycohemoglobin standardization program, CVD: cardiovascular disease (including stroke).

**Table 2:** Odds ratios of each sleep duration for hearing loss.

Frequency	Sleep durations	≤5 h	6 h	7 h	8 h	≥9 h
4,000 Hz						
Model 1		1	1.27 (1.10–1.46)^‡^	2.06 (1.79–2.36)^‡^	3.92 (3.39–4.54)^‡^	8.38 (6.50–10.8)^‡^
Model 2	a	1	1.04 (0.90–1.21)	1.17 (1.01–1.37)∗	1.38 (1.17–1.64)^‡^	1.82 (1.32–2.50)^‡^
	b	0.98 (0.84–1.14)	1	1.11 (1.01–1.21)∗	1.30 (1.16–1.45)^‡^	1.72 (1.28–2.31)^‡^
Model 3		1	1.07 (0.90–1.27)	1.20 (1.01–1.43)∗	1.36 (1.13–1.64)^†^	1.75 (1.22–2.50)^†^
1,000 Hz						
Model 1		1	1.08 (0.89–1.31)	1.42 (1.17–1.73)^‡^	2.41 (1.96–2.97)^‡^	6.80 (4.88–9.48)^‡^
Model 2	a	1	0.93 (0.77–1.14)	0.95 (0.77–1.16)	1.13 (0.90–1.41)	2.06 (1.43–2.97)^‡^
	b	1.07 (0.88–1.31)	1	1.02 (0.90–1.15)	1.21 (1.04–1.41)∗	2.21 (1.60–3.07)^‡^
Model 3		1	1.00 (0.80–1.24)	1.00 (0.79–1.25)	1.16 (0.90–1.49)	2.20 (1.47–3.29)^‡^

*P < 0.05, ^†^ *P* < 0.01, and ^‡^ *P* < 0.001.

The number of subjects in each group is the same as that in Table 1.

Hearing loss was defined as >25 dB hearing level in the right and/or left sides of ear.

Model 1: unadjusted.

Model 2: adjusted for age, sex, smoking, alcohol consumption, and having regular exercise, quartile of white blood cell counts, six tiles of body mass index, and past history of cardiovascular disease, complications (hypertension, dyslipidemia, and diabetes), self-reported tinnitus, working duration, and occupation.

The reference sleep duration was ≤5 h in Model 2a and 6 h in Model 2b.

Model 3: Model 2a plus adjustments for organic solvent work (available *n* = 39,691).

**Table 3:** Odds ratio of cardiovascular risk factors for hearing loss.

Models	4,000 Hz		1,000 Hz	
	Unadjusted	Multivariate adjusted	Unadjusted	Multivariate adjusted
Gender				
Men (versus women)	5.69 (5.07–6.37)^‡^	4.47 (3.87–5.15)^‡^	1.22 (1.09–1.36)^‡^	1.18 (1.01–1.37)∗
Body mass index six categories				
≤19.0 kg/m^2^	0.73 (0.63–0.86)^‡^	1.22 (1.02–1.47)∗	0.98 (0.79–1.20)	1.23 (0.99–1.53)
19.1–21.0 kg/m^2^	0.80 (0.71–0.89)^‡^	1.06 (0.93–1.20)	0.81 (0.69–0.96)∗	0.94 (0.80–1.11)
21.1–22.9 kg/m^2^	1	1	1	1
23.0–24.9 kg/m^2^	1.37 (1.25–1.50)^‡^	1.05 (0.94–1.17)	1.05 (0.91–1.21)	0.89 (0.77–1.03)
25.0–26.9 kg/m^2^	1.46 (1.32–1.62)^‡^	1.06 (0.95–1.19)	1.10 (0.94–1.28)	0.89 (0.76–1.05)
≥27.0 kg/m^2^	1.00 (0.90–1.11)	0.95 (0.84–1.08)	0.88 (0.74–1.03)	0.88 (0.74–1.05)
White blood cell				
<25% tile	1	1	1	1
25–49.9% tile	1.29 (1.17–1.42)^‡^	1.08 (0.96–1.21)	1.15 (1.00–1.33)	1.10 (0.95–1.28)
50–75% tile	1.38 (1.25–1.52)^‡^	1.07 (0.95–1.19)	1.13 (0.98–1.31)	1.07 (0.92–1.24)
>75% tile (≥88.9 × 10^2^/*μ*L)	1.67 (1.52–1.83)^‡^	1.15 (1.03–1.29)∗	1.18 (1.02–1.36)∗	1.08 (0.92–1.26)
Past history of cardiovascular disease^a,b^	2.08 (1.70–2.55)^‡^	1.08 (0.85–1.36)	1.91 (1.40–2.58)^‡^	1.15 (0.83–1.58)
Complication of				
Hypertension^b^	3.14 (2.85–3.45)^‡^	1.00 (0.90–1.11)	2.46 (2.15–2.81)^‡^	1.07 (0.92–1.23)
Dyslipidemia^b^	1.27 (1.10–1.46)^†^	0.81 (0.70–0.95)^†^	1.51 (1.24–1.84)^‡^	1.00 (0.82–1.23)
Diabetes^b^	3.71 (3.27–4.21)^‡^	1.53 (1.32–1.76)^‡^	3.06 (2.54–3.68)^‡^	1.56 (1.28–1.90)^‡^
Tinnitus				
Present^b^	4.51 (3.99–5.09)^‡^	3.13 (2.71–3.62)^‡^	3.13 (2.60–3.77)^‡^	2.03 (1.67–2.47)^‡^
Smoking				
No smoker	1	1	1	1
Past smoker	1.89 (1.71–2.09)^‡^	0.98 (0.87–1.11)	1.19 (1.02–1.39)	0.96 (0.81–1.13)
Current smoker	2.04 (1.90–2.18)^‡^	1.29 (1.18–1.41)^‡^	1.15 (1.04–1.28)^†^	1.08 (0.95–1.22)
Alcohol consumption				
No drinker	1	1	1	1
Occasional drinker	0.54 (0.48–0.60)^‡^	0.95 (0.84–1.07)	0.49 (0.42–0.56)^‡^	0.87 (0.74–1.01)
1~3/week drinker	0.77 (0.69–0.86)^‡^	0.85 (0.75–0.96)^†^	0.62 (0.52–0.73)^‡^	0.85 (0.72–1.01)
4~6/week drinker	1.31 (1.18–1.46)^‡^	0.93 (0.83–1.06)	0.82 (0.70–0.97)∗	0.82 (0.69–0.98)∗
Daily drinker	2.40 (2.21–2.62)^‡^	1.17 (1.05–1.30)^†^	1.37 (1.20–1.55)^‡^	1.04 (0.90–1.20)
Exercise				
≥2/week exerciser	1	1	1	1
1/week exerciser	0.94 (0.84–1.05)	0.99 (0.87–1.13)	0.83 (0.71–0.99)∗	0.93 (0.78–1.10)
Occasional exerciser	0.88 (0.78–0.99)∗	1.12 (0.97–1.29)	0.72 (0.59–0.87)^‡^	0.99 (0.81–1.21)
No exerciser	1.13 (1.03–1.23)∗	1.18 (1.06–1.31)^†^	1.10 (0.96–1.25)	1.18 (1.02–1.36)∗
Work duration				
≤6 h	1	1	1	1
7 h	1.58 (1.34–1.85)^‡^	1.05 (0.86–1.29)	0.89 (0.73–1.10)	0.90 (0.72–1.11)
8 h	1.27 (1.12–1.45)^‡^	1.00 (0.85–1.18)	0.61 (0.52–0.70)^‡^	0.85 (0.71–1.01)
9 h	0.96 (0.83–1.11)	0.87 (0.72–1.06)	0.45 (0.37–0.55)^‡^	0.76 (0.61–0.95)∗
10 h	0.93 (0.79–1.08)	0.92 (0.76–1.11)	0.40 (0.32–0.49)^‡^	0.71 (0.56–0.90)^†^
≥11 h	0.70 (0.60–0.82)^‡^	0.87 (0.72–1.06)	0.29 (0.24–0.36)^‡^	0.62 (0.48–0.79)^‡^

*P < 0.05, ^†^ *P* < 0.01, and ^‡^ *P* < 0.001.

^a^Cardiovascular disease including heart disease and stroke.

^b^Present versus absent.

Confounding factors in multivariate adjustments include those listed in Model 2a of Table 2.

Numbers in exercise express the times of exercise (>30 min per session).

**Table 4:** Baseline clinical characteristics of subjects and prevalence of longer sleep duration eight years later in the longitudinal study.

Categories by sleep duration	Total	≤5 h	6 h	7 h	*P* values
*N*, (% of total)	6,774	450 (10.5)	3,063 (46.6)	3,261 (31.4)	—
Age (years)	41.6 ± 9.0	40.2 ± 9.5	41.2 ± 9.1	42.1 ± 8.8	<0.0001
Men, *n* (%)	4,639 (68.5)	268 (59.6)	1,959 (64.0)	2,412 (74.0)	<0.0001
BMI (kg/m^2^)	23.2 ± 3.3	23.3 ± 3.6	23.3 ± 3.4	23.2 ± 3.1	0.67
Systolic blood pressure (mmHg)	123 ± 16.0	122 ± 15.7	123 ± 16.0	124 ± 16.0	<0.0001
Diastolic blood pressure (mmHg)	74 ± 12.2	72 ± 12.2	74 ± 12.2	75 ± 12.1	<0.0001
HbA1c (%, NGSP)	5.4 ± 0.6	5.4 ± 0.8	5.4 ± 0.6	5.4 ± 0.6	0.39
White blood cell (×10^2^/*μ*L)	65.0 ± 17.4	64.0 ± 16.4	64.9 ± 17.7	65.3 ± 17.1	0.32
Past history of CVD, *n* (%)	84 (1.2)	10 (2.2)	36 (1.2)	38 (1.2)	0.15
Medication for					
Hypertension, *n* (%)	259 (3.8)	13 (2.9)	113 (3.7)	133 (4.1)	0.41
Dyslipidemia, *n* (%)	71 (1.0)	0 (0.0)	33 (1.1)	38 (1.2)	—∗
Diabetes, *n* (%)	66 (1.0)	5 (1.1)	32 (1.0)	29 (0.9)	—∗
Alcohol consumption					
No~occasional/1~3/w/4~6/w/daily (%)∗	45/23/11/22	48/26/8/17	48/23/11/18	41/22/12/26	<0.0001
Current smoker, *n* (%)	2,606 (38.5)	171 (38.0)	1,120 (36.6)	1,315 (40.3)	0.009
Having regular exercise					
No/occasional/1/w/≥2/w (%)∗	50/13/18/20	54/14/14/18	50/13/18/20	47/14/19/20	0.09
Hearing loss at least in one ear at 4,000 Hz, *n* (%)	457 (6.7)	19 (4.2)	175 (5.7)	263 (8.1)	<0.0001
Hearing loss at least in one ear at 1,000 Hz, *n* (%)	168 (2.5)	8 (1.8)	77 (2.5)	83 (2.5)	0.61
Work duration (hour) (available *n* = 6,703)	8.5 ± 1.2	8.9 ± 1.4	8.6 ± 1.2	8.4 ± 1.1	<0.0001
Prevalence of longer sleep duration eight years later, *n* (%)	452 (6.7)	11 (2.4)	109 (3.6)	332 (10.2)	<0.0001

The data are expressed as means ± SD. Triglyceride is expressed as medians (interquartiles).

Individuals with longer sleep duration (≥8 hour) at baseline were not enrolled in the longitudinal study.

∗Total sum is not 100% in some cases because of decimal points.

BMI: body mass index, HDL: high-density lipoprotein, NGSP: national glycohemoglobin standardization program, and CVD: cardiovascular disease (including stroke).

**Table 5:** Relative risks for longer sleep duration according to baseline hearing loss.

	Baseline hearing loss at 4000 Hz		Baseline hearing loss at 1000 Hz	
	Absent	Present	Absent	Present
Baseline	6317	457	6606	168
Long-time sleep in 2008	394	58	433	19

	Relative risk (95% CI)		Relative risk (95% CI)	

Model 1	1	2.19 (1.63–2.93)^†^	1	1.82 (1.12–2.96)∗
Model 2	1	1.56 (1.14–2.13)∗	1	1.50 (0.92–2.47)
Model 3	1	1.53 (1.11–2.10)∗	1	1.52 (0.92–2.50)

*P < 0.01 and ^†^ *P* < 0.001.

Longer sleep duration at the time point of 9 years was defined as ≥8 h. Hearing loss was defined as loss of hearing of >25 dB in at least one ear.

The number of subjects in each group is the same as that in Table 3.

Model 1: unadjusted.

Model 2: adjusted for age, sex, smoking, alcohol consumption, and regular exercise.

Model 3: Model 2 plus baseline medications for hypertension, dyslipidemia, and diabetes; baseline past history of cardiovascular disease; and baseline body mass index and white blood cell count (total available *n* = 6250).

## References

[1] Howarth A, Shone GR (2006). Ageing and the auditory system. *Postgraduate Medical Journal*.

[2] Liu XZ, Yan D (2007). Ageing and hearing loss. *Journal of Pathology*.

[3] Huang Q, Tang J (2010). Age-related hearing loss or presbycusis. *European Archives of Oto-Rhino-Laryngology*.

[4] Li-Korotky HS (2012). Age-related hearing loss: quality of care for quality of life. *Gerontologist*.

[5] Ciorba A, Bianchini C, Pelucchi S, Pastore A (2012). The impact of hearing loss on the quality of life of elderly adults. *Clinical Interventions in Aging*.

[6] Moyer VA, U.S. Preventive Services Task Force (2012). Screening for hearing loss in older adults: U.S. preventive services task force recommendation statement. *Annals of Internal Medicine*.

[7] Hallam RS (1996). Correlates of sleep disturbance in chronic distressing tinnitus. *Scandinavian Audiology*.

[8] Asplund R (2003). Sleepiness and sleep in elderly subjects with hearing complaints. *Archives of Gerontology and Geriatrics*.

[9] Hume KI (2011). Noise pollution: a ubiquitous unrecognized disruptor of sleep?. *Sleep*.

[10] Test T, Canfi A, Eyal A, Shoam-Vardi I, Sheiner EK (2011). The influence of hearing impairment on sleep quality among workers exposed to harmful noise. *Sleep*.

[11] Ito K, Naito R, Murofushi T, Iguchi R (2007). Questionnaire and interview in screening for hearing impairment in adults. *Acta Oto-laryngologica*.

[12] Nakanishi N, Okamoto M, Nakamura K, Suzuki K, Tatara K (2000). Cigarette smoking and risk for hearing impairment: a longitudinal study in Japanese male office workers. *Journal of Occupational and Environmental Medicine*.

[13] Takata Y (2011). Hearing loss associated with smoking in male workers. *Journal of UOEH*.

[14] Kakarlapudi V, Sawyer R, Staecker H (2003). The effect of diabetes on sensorineural hearing loss. *Otology and Neurotology*.

[15] Austin DF, Konrad-Martin D, Griest S, McMillan GP, McDermott D, Fausti S (2009). Diabetes-related changes in hearing. *Laryngoscope*.

[16] Bainbridge KE, Cheng YJ, Cowie CC (2010). Potential mediators of diabetes-related hearing impairment in the U.S. population: National Health and Nutrition Examination Survey 1999–2004. *Diabetes Care*.

[17] Muneyuki T, Sugawara H, Suwa K (2013). Design of the Saitama Cardiometabolic Disease and Organ Impairment Study (SCDOIS): a multidisciplinary observational epidemiological study. *Open Journal of Endocrine and Metabolic Diseases*.

[18] Saitama Health Promotion Corporation http://www.saitama-kenkou.or.jp/.

[19] Tsuno N, Besset A, Ritchie K (2005). Sleep and depression. *Journal of Clinical Psychiatry*.

[20] Kaneita Y, Ohida T, Uchiyama M (2006). The relationship between depression and sleep disturbances: a Japanese nationwide general population survey. *Journal of Clinical Psychiatry*.

[21] Mezick EJ, Hall M, Matthews KA (2011). Are sleep and depression independent or overlapping risk factors for cardiometabolic disease?. *Sleep Medicine Reviews*.

[22] Loredo JS, Soler X, Bardwell W, Ancoli-Israel S, Dimsdale JE, Palinkas LA (2010). Sleep health in U.S. Hispanic population. *Sleep*.

[23] Kashiwagi A, Kasuga M, Araki E (2012). International clinical harmonization of glycated hemoglobin in Japan: from Japan Diabetes Society to National Glycohemoglobin Standardization Program values. *Journal of Diabetes Investigation*.

[24] Sabanayagam C, Shankar A (2010). Sleep duration and cardiovascular disease: results from the National Health Interview Survey. *Sleep*.

[25] Shankar A, Charumathi S, Kalidindi S (2011). Sleep duration and self-rated health: the National Health Interview Survey 2008. *Sleep*.

[26] WHO Expert Consultation (2004). Appropriate body-mass index for Asian populations and its implications for policy and intervention strategies. *The Lancet*.

[27] Weijenberg MP, Feskens EJM, Kromhout D (1996). White blood cell count and the risk of coronary heart disease and all-cause mortality in elderly men. *Arteriosclerosis, Thrombosis, and Vascular Biology*.

[28] Sun HJ, Jung YP, Kim H, Tae YL, Samet JM (2005). White blood cell count and risk for all-cause, cardiovascular, and cancer mortality in a cohort of Koreans. *The American Journal of Epidemiology*.

[29] Jia E, Yang Z, Yuan B (2005). Relationship between leukocyte count and angiographical characteristics of coronary atherosclerosis. *Acta Pharmacologica Sinica*.

[30] Kiesswetter E, Seeber A, Nat R, Golka K, Sietmann B (1997). Solvent exposure, shiftwork, and sleep. *International Journal of Occupational and Environmental Health*.

[31] Viaene M, Vermeir G, Godderis L (2009). Sleep disturbances and occupational exposure to solvents. *Sleep Medicine Reviews*.

[32] Zocchetti C, Consonni D, Bertazzi PA (1997). Relationship between prevalence rate ratios and odds ratios in cross-sectional studies. *International Journal of Epidemiology*.

[33] Knutson KL (2010). Sleep duration and cardiometabolic risk: a review of the epidemiologic evidence. *Best Practice and Research: Clinical Endocrinology and Metabolism*.

[34] Chao C, Wu J, Yang Y (2011). Sleep duration is a potential risk factor for newly diagnosed type 2 diabetes mellitus. *Metabolism: Clinical and Experimental*.

[35] Hong O (2005). Hearing loss among operating engineers in American construction industry. *International Archives of Occupational and Environmental Health*.

[36] Bergström B, Nyström B (1986). Development of hearing loss during long-term exposure to occupational noise. A 20-year follow-up study. *Scandinavian Audiology*.

[37] Mostaghaci M, Mirmohammadi SJ, Mehrparvar AH, Bahaloo M, Mollasadeghi A, Davari MH (2013). Effect of workplace noise on hearing ability in tile and ceramic industry workers in Iran: a 2-year follow-up study. *The Scientific World Journal*.

[38] Williams CJ, Hu FB, Patel SR, Mantzoros CS (2007). Sleep duration and snoring in relation to biomarkers of cardiovascular disease risk among women with type 2 diabetes. *Diabetes Care*.

[39] Wallhagen MI, Strawbridge WJ, Shema SJ (2008). The relationship between hearing impairment and cognitive function: a 5-year longitudinal study. *Research in Gerontological Nursing*.

[40] Lin FR, Ferrucci L, Metter EJ, An Y, Zonderman AB, Resnick SM (2011). Hearing loss and cognition in the Baltimore Longitudinal Study of Aging. *Neuropsychology*.

[41] Xu L, Jiang CQ, Lam TH (2011). Short or long sleep duration is associated with memory impairment in older chinese: the Guangzhou Biobank Cohort Study. *Sleep*.

[42] Lin FR, Yaffe K, Xia J (2013). Hearing loss and cognitive decline in older adults. *JAMA Internal Medicine*.

[43] Most EI, Aboudan S, Scheltens P, van Someren EJ (2012). Discrepancy between subjective and objective sleep disturbances in early- and moderate-stage Alzheimer disease. *The American Journal of Geriatric Psychiatry*.

[44] Mazzotti DR, Guindalini C, Sosa AL, Ferri CP, Tufik S (2012). Prevalence and correlates for sleep complaints in older adults in low and middle income countries: a 10/66 Dementia Research Group study. *Sleep Medicine*.

[45] Ju YS, McLeland JS, Toedebusch CD (2013). Sleep quality and preclinical Alzheimer disease. *JAMA Neurology*.

[46] Fruhstorfer B, Pritsch MG, Fruhstorfer H (1988). Effects of daytime noise load on the sleep-wake cycle and endocrine patterns in man: I. 24 hours neurophysiological data. *International Journal of Neuroscience*.

[47] Abad-Alegría F, Gutiérrez M (1998). Characteristics of sleep in deafness. *Revue Neurologique*.

[48] Rios AL, Alves da Silva G (2005). Sleep quality in noise exposed Brazilian workers. *Noise and Health*.

[49] Wackym PA, Linthicum FH (1986). Diabetes mellitus and hearing loss: clinical and histopathologic relationships. *The American Journal of Otology*.

[50] Smith TL, Raynor E, Prazma J, Buenting JE, Pillsbury HC (1995). Insulin-dependent diabetic microangiopathy in the inner ear. *Laryngoscope*.

[51] Shemesh Z, Attias J, Ornan M, Shapira N, Shahar A (1993). Vitamin B12 deficiency in patients with chronic-tinnitus and noise-induced hearing loss. *American Journal of Otolaryngology—Head and Neck Medicine and Surgery*.

[52] Houston DK, Johnson MA, Nozza RJ (1999). Age-related hearing loss, vitamin B-12, and folate in elderly women. *American Journal of Clinical Nutrition*.

[53] Shargorodsky J, Curhan SG, Eavey R, Curhan GC (2010). A prospective study of vitamin intake and the risk of hearing loss in men. *Otolaryngology—Head and Neck Surgery*.

[54] Lasisi AO, Fehintola FA, Yusuf OB (2010). Age-related hearing loss, vitamin B12, and folate in the elderly. *Otolaryngology-Head and Neck Surgery*.

[55] Gopinath B, Flood VM, McMahon CM (2011). Dietary antioxidant intake is associated with the prevalence but not incidence of age-related hearing loss. *Journal of Nutrition, Health and Aging*.

[56] Jee SH, Sull JW, Park J, Lee SY, Ohrr H, Guallar E (2006). Body-mass index and mortality in Korean men and women. *The New England Journal of Medicine*.

[57] de Gonzalez AB, Hartge P, Cerhan JR (2010). Body-mass index and mortality among 1.46 million white adults. *The New England Journal of Medicine*.

[58] Zheng W, McLerran DF, Rolland B (2011). Association between body-mass index and risk of death in more than 1 million Asians. *The New England Journal of Medicine*.

[59] Rehm J, Mathers C, Popova S, Thavorncharoensap M, Teerawattananon Y, Patra J (2009). Global burden of disease and injury and economic cost attributable to alcohol use and alcohol-use disorders. *The Lancet*.

[60] Ronksley PE, Brien SE, Turner BJ, Mukamal KJ, Ghali WA (2011). Association of alcohol consumption with selected cardiovascular disease outcomes: a systematic review and meta-analysis. *BMJ*.

[61] Iso H (2011). Lifestyle and cardiovascular disease in Japan. *Journal of Atherosclerosis and Thrombosis*.

[62] Gupta R, Deedwania P (2011). Interventions for cardiovascular disease prevention. *Cardiology Clinics*.

[63] Udo Y, Nakao M, Honjo H, Ukimura O, Kitakoji H, Miki T (2009). Sleep duration is an independent factor in nocturia: analysis of bladder diaries. *BJU International*.

[64] Cohen-Mansfield J, Perach R (2012). Sleep duration, nap habits, and mortality in older persons. *Sleep*.

[65] Ryu SY, Kim KS, Han MA (2011). Factors associated with sleep duration in Korean adults: Results of a 2008 community health survey in Gwangju Metropolitan City, Korea. *Journal of Korean Medical Science*.

[66] Ertel KA, Berkman LF, Buxton OM (2011). Socioeconomic status, occupational characteristics, and sleep duration in African/Caribbean immigrants and US white health care workers. *Sleep*.

[67] Lallukka T, Sares-Jäske L, Kronholm E (2012). Sociodemographic and socioeconomic differences in sleep duration and insomnia-related symptoms in Finnish adults. *BMC Public Health*.

[68] Chou R, Dana T, Bougatsos C, Fleming C, Beil T (2011). Screening adults aged 50 years or older for hearing loss: a review of the evidence for the U.S. preventive services task force. *Annals of Internal Medicine*.

